# Long-Range Network of Air Quality Index Sensors in an Urban Area

**DOI:** 10.3390/s23219001

**Published:** 2023-11-06

**Authors:** Ionut-Marian Dobra, Vladut-Alexandru Dobra, Adina-Alexandra Dobra, Gabriel Harja, Silviu Folea, Vlad-Dacian Gavra

**Affiliations:** 1 Faculty of Automatic Control and Computer Science, Technical University of Cluj-Napoca, 400114 Cluj-Napoca, Romania; ionut.dobra@aut.utcluj.ro (I.-M.D.); vlad.dobra@aut.utcluj.ro (V.-A.D.); gabriel.harja@aut.utcluj.ro (G.H.); silviu.folea@aut.utcluj.ro (S.F.); 2 Faculty of Industrial Engineering, Robotics and Production Management, Technical University of Cluj-Napoca, 400114 Cluj-Napoca, Romania; adina.bretan@muri.utcluj.ro; 3 Faculty of Electronics, Telecommunications and Information Technology, Technical University of Cluj-Napoca, 400114 Cluj-Napoca, Romania

**Keywords:** IoT (Internet of Thing), AQI (Air Quality Index), LoRa (long range), pollution map

## Abstract

In recent times the escalating pollution within densely populated metropolitan areas has emerged as a significant and pressing concern. Authorities are actively grappling with the challenge of devising solutions to promote a cleaner and more environmentally friendly urban landscapes. This paper outlines the potential of establishing a LoRa node network within a densely populated urban environment. Each LoRa node in this network is equipped with an air quality measurement sensor. This interconnected system efficiently transmits all the analyzed data to a gateway, which subsequently sends it to a server or database in real time. These data are then harnessed to create a pollution map for the corresponding area, providing users with the opportunity to assess local pollution levels and their recent variations. Furthermore, this information proves valuable when determining the optimal route between two points in the city, enabling users to select the path with the lowest pollution levels, thus enhancing the overall quality of the urban environment. This advantage contributes to alleviating congestion and reducing excessive pollution often concentrated behind buildings or on adjacent streets.

## 1. Introduction

Pollution, in general, can lead to a range of health issues, some of which can become severe when individuals are exposed to polluted environments for extended periods. Pollution remains a major global health threat, and the responsibility lies with us to enhance our work and living environments. Potential problems stemming from pollution include respiratory and cardiovascular issues, asthma, allergies, nausea, reduced concentration, heart attacks, spontaneous abortions, difficulties with focus, and even the development of cancer.

Currently, nitrogen oxide emissions result from vehicle exhaust, coal-fired power plants, and various discharges from industrial facilities. Conversely, VOCs are generated through the use of gasoline, cleaning products, and paints. When these two substances combine with sunlight, they create ground-level ozone and smog.

The environment significantly shapes our daily work and life, making air quality in the area a vital factor that profoundly affects our lives. To gain control over this aspect, it is imperative to conduct measurements and establish reference points to enhance our overall well-being.

During the COVID-19 pandemic lockdown, air pollution witnessed a significant reduction. The strict confinement measures prohibited individuals from going outside, resulting in nearly nonexistent traffic. Furthermore, major factories and industries were temporarily suspended to mitigate the risk of virus transmission in communal spaces. It became evident to all how these activities had a substantial impact on the climate and inadvertently led to improvements [1,2,3,4].

When examining surveillance data from two streets in Copenhagen, the contention was that readings obtained near roadways were highly influenced by their specific locations and did not offer a representative view of air quality across a broader urban area. This highlighted the considerable disparities in measured pollutant concentrations between these two sites. Another study emphasized the significant impact of local wind patterns and the spatial configuration of streets and buildings surrounding a measurement point on air quality readings at the juncture of two central London streets.

The total count of fixed air quality monitoring stations within a city is inherently limited due to practical constraints, including factors such as equipment cost, size, and power requirements. Expanding the number of fixed stations is often a challenging endeavor. Consequently, alternative measurement and modeling techniques are essential for evaluating urban air quality in locations where monitoring stations are not present [5,6].

There are also publications that describe an air pollution mapping with a sensor-based methodology by using a drone with only one setup to analyze the environment in different locations [7]. The mentioned setup is not robust enough from two points of view. The first problem with a setup like this is that a drone is creating a displacement of a huge volume of air meaning that the results are not accurate. The second point is that the drone can only cover a specific zone in a defined time frame, and this is not possible in any meteorologic condition. After the analysis is done, the drone needs to return to the home point and to charge for a relatively big amount of time compared to the amount of time spent to gather data. From this point of view, the setup using a drone is not feasible for this type of measurement.

All of us collectively hold the aspiration to navigate a specific urban area with minimal pollution and to access pollution data for that region. Regrettably, at present, such a pollution map is not available. To bridge this gap, the suggestion is to establish a network of air quality sensors. These sensors would possess the capability to measure pollution levels and transmit this data via the LoRa network to a central server.

This process would enable the creation of a pollution map for the designated neighborhood or city in real time. This real-time pollution map would provide individuals with the information they need to make informed decisions. For example, they could assess whether it is advisable to go outdoors based on the current pollution levels. If they decide to venture outside, the map would also help in identifying areas with lower pollution levels and suggest the best routes, ultimately promoting better health protection.

The primary objective of this sensor network is to gather air quality data, centralize it, process it effectively, and present it visually in real time. This ensures that individuals seeking information about current air quality can readily comprehend potential risks without the need for complex calculations [8,9].

To effectively establish this sensor network, the project will be divided into three distinct components. Firstly, an exploration of the existing research efforts focused on identifying specific environmental parameters that necessitate outdoor measurement will be conducted. This will particularly emphasize those crucial in assessing air pollution. Additionally, there will be an examination of the selection of an optimal data transmission technology for the application, with a specific emphasis on evaluating the suitability of LoRa technology [10].

The second segment of the project will encompass a comprehensive presentation of the approach taken to deploy the sensor network within a neighborhood. The rationale behind this deployment, including anticipated benefits and acknowledgment of potential limitations, will be elucidated. Moreover, insights into the network’s configuration and an explanation of the data procurement procedures will be provided.

In the final section of the document, an explanation will be given on how this sensor network, through the collection and centralization of data into a database, can play a pivotal role in determining the cleanest route based on air quality considerations. The concluding part will include the study’s findings, detailing the practical achievements and hardware and software system limitations. Additionally, it will present future implementations or forthcoming studies. These additional implementations involve the development of new hardware and software with reduced dimensions and lowered expenses, enabling the sensor mesh to be deployed effectively within a densely populated urban environment. This cost-effective approach allows for the deployment of over 1000 end nodes, ensuring comprehensive coverage of highly frequented areas.

## 2. Materials and Methods

### 2.1. Overview of LoRa Technology

The LoRa Alliance is a nonprofit organization that operates in an open and collaborative manner. Since its establishment in 2015, it has grown rapidly and is now one of the largest and fastest-growing alliances in the technology sector. Its members work closely together, sharing their experiences and efforts to support and advance the LoRaWAN standard, which is recognized as the leading worldwide standard for secure IoT LPWAN (Low Power Wide Area Network) connectivity. LoRaWAN offers the technical versatility to cater to a wide array of IoT applications [11], both stationary and mobile. Furthermore, it has a certification program in place to ensure seamless interoperability. Major mobile network operators around the world have already implemented LoRaWAN^®^, and this adoption is expected to continue expanding significantly in 2023 and beyond [12].

The primary objective of the LoRa Alliance^®^ is to establish standardized LPWAN technology, with the overarching goal of facilitating extensive IoT deployments on a large scale. This standardization paves the way for widespread availability of IoT products, while the LoRaWAN^®^ Certification Program guarantees seamless interoperability. These achievements are the direct result of our members’ collaborative efforts in developing and refining the LoRaWAN^®^ standard.

The primary IoT applications that rely on LPWA (Low Power Wide Area) technology necessitate extended battery life to support devices that can be deployed and essentially forgotten or disposed of, a cost-effective sensor or device bill of materials (BOM), and the ability to establish long-range connectivity [13].

While the potential applications for LPWAN technology are vast, the driving forces behind current network deployments predominantly revolve around intelligent building solutions, supply chain management, Smart City initiatives, and agriculture.

In the realm of intelligent building solutions, the primary value proposition lies in insurance cost reduction and efficient servicing. For instance, in regions prone to cold weather, a ruptured water pipe can lead to insurance claims of approximately USD 50,000. Insurance companies often offer premium discounts when building management solutions are implemented. Utilizing sensors to determine whether buildings or rooms are in use can significantly reduce expenses related to service management.

In supply chain management, any application involving deliveries, pick-ups, and inventory management stands to benefit from LPWAN technology. It enables substantial savings through inventory management and optimization of delivery routes. For instance, a smart trash monitoring solution can reduce pick-up frequency by up to 40%.

In agriculture, the driving factors are rooted in the increasing demand for food production. Agriculture accounts for 80% of water usage, and crops have high value. Therefore, the deployment of sensors to monitor water usage and the health of soil and crops becomes crucial. Precise irrigation and soil monitoring result in significant cost savings in resource utilization and lead to improved profits through enhanced yields.

The LoRa WAN protocol [14] is a relatively recent communication protocol designed to establish cost-effective and dependable wireless connections across various IoT (Internet of Things) scenarios. Operating as a “Low Power Wide Area Network” technology within the ISM band, it gained rapid popularity, finding utility both in industrial and academic environments.

As defined by the LoRa Alliance [15], LoRa stands for “Low-Range”, “Low-Power”, and “Low-Data-Rate” applications, signifying its role in facilitating wireless communication for such purposes. The LoRa network is further categorized into different transmission classes: LoRaWAN basic transmission, also known as Class A, and secondary transmissions, encompassing Class B and Class C.

Class A, or end devices with bidirectional communication capabilities, allows for two-way communication where each end device, following an “uplink” transmission, follows up with two brief “downlink” windows. The timing of these transmission slots is determined by the end devices based on their communication requirements, with slight variations introduced through random time programming (similar to an ALOHA-type protocol). Class A operations are characterized by the lowest power consumption among end devices in the system and are suitable for applications that primarily necessitate downlink transmissions. Downlink communications from the server can only occur during the next scheduled uplink interval.

Class B, or end devices with two-way communication featuring programmed receive slots, enables these devices to open multiple receiving slots. In addition to receiving random windows like Class A, Class B devices can initiate additional receiving windows at specified times. To synchronize their receive windows with precise timing, Class B devices receive a synchronization signal from the gateway.

Class C, or end devices with two-way communication and a maximum number of slots, have their receive windows nearly continuously open, closing them only when transmitting. Class C devices consume more power compared to Class A or Class B devices but offer the shortest latency in server communication with end devices [16].

The LoRa modulation technique is based on variations of “Spread Spectrum Modulation” and “Chirp Spread Spectrum (CSS)”, incorporating error correction mechanisms in the transmission process.

Benefits of employing LoRa technology [17,18]:

1. Extended Coverage—establishes connections spanning distances of up to 48 km in rural areas, while in urban environments, it excels at penetrating dense or congested indoor spaces.
2. Energy Efficiency—demands minimal energy, resulting in extended battery life of up to 10 years, thereby reducing the costs associated with battery replacement.
3. Security—offers end-to-end functionality featuring robust AES128 encryption, mutual authentication, integrity protection, and privacy measures.
4. Standardization—ensures device interoperability and the global availability of LoRaWAN networks, facilitating swift deployment in IoT applications across diverse locations.
5. Geolocation—permits applications to determine device locations without relying on GPS, delivering unique low-power advantages not achievable with other technologies.
6. Mobility—sustains communication with devices in motion while maintaining energy efficiency, requiring minimal power resources.
7. High Capacity—enables the transmission of millions of messages per base station, meeting the demands of public network operators serving extensive markets.
8. Cost Reduction—decreases infrastructure investment costs, lowers expenses associated with battery replacements, and incurs minimal operational costs.

**Figure 1:**
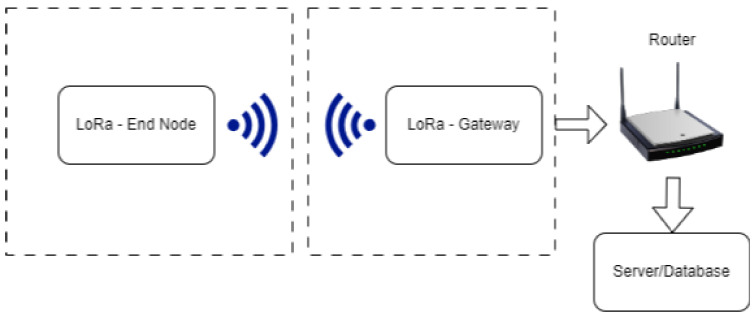
Communication between end node–gateway–server.

LoRaWAN as the communication is presented in Figure 1 is used in a very wide area including smart cities, industrial monitoring, smart building, and agriculture. In the context of the smart city, it is used for efficient monitoring of resources such as lighting, parking lots and environmental conditions. In industrial environments, LoRaWAN facilitates remote monitoring of equipment, optimization of supply chains and predictive maintenance. Agriculture benefits from LoRaWAN through livestock tracking, soil, and irrigation monitoring. LoRaWAN smart buildings for tasks such as occupancy detection, monitoring security systems, and energy management.

LoRaWAN also plays a critical role in environmental monitoring, including air and water quality assessments.

### 2.2. Used Equipment

For the measurements a set of devices were used where the code was created, configured and fine tunned to be able to achieve the best results. The devices used for the data transmission via LoRa protocol are the from ST Microcontroller. ST LoRa (Long-Range) communication typically operates within a LoRaWAN (Long-Range Wide Area Network) architecture, which involves communication between devices (such as STM32WL-based nodes) and gateways, followed by communication from gateways to a central server.

The communication is done in the following way:

I. Device to Gateway: ST LoRa devices (like the STM32WL) use LoRa modulation to send data to nearby LoRa gateways. These gateways are equipped with LoRa receivers and are strategically placed to cover a specific geographical area. Devices transmit data using the LoRa modulation, and gateways with appropriate receivers pick up these transmissions.

**Figure 2:**
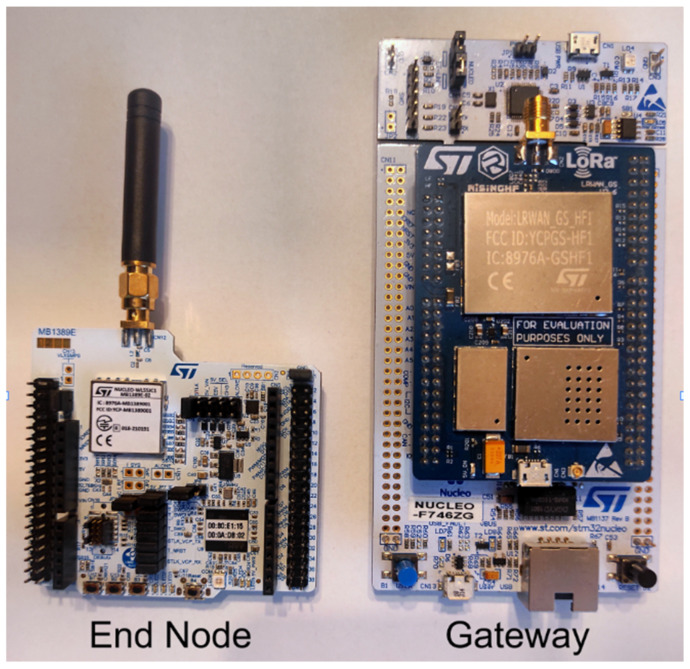
End node: NUCLEO-WL55JC1 (**left** side) and gateway: NUCLEO-F746ZG (**right** side).

The end node is a NUCLEO-WL55JC1 board [19] that can be observed in Figure 2 on the left side, that is able to communicate on the 868 MHz frequency with the gateway and able to harness the information from the environment (like the temperature, humidity ore air quality) with the connected Air Quality sensor BME680 from Bosch.

The configuration of the end node was initiated by using the Nucleo WL55JC1 demo provided by ST Microcontroller as a foundation, with subsequent software adjustments tailored to the specific use case. In the initial setup, specific modifications were required for each end node to establish communication.

1. LORAWAN_DEVICE_EUI—configured with the unique identifier from each node.
2. LORAWAN_JOIN_EUI—left as default so the end node could join any existing gateway in their proximity and transmit data to the TTN (the things network)
3. LORAWAN_APP_KEY—generate for each end node to be different.
4. LORAWAN_NWK_KEY—can be the same as LORAWAN_APP_KEY for the current prototype testing.
5. LORAWAN_NWK_S_KE—can be the same as LORAWAN_APP_KEY for the current prototype testing.
6. LORAWAN_APP_S_KEY—can be the same as LORAWAN_APP_KEY for the current prototype testing.

After configuring the end node in the software, it is necessary to define the active region. Given that the tests take place in Europe, the frequency plan operates at 868 MHz, making it essential to activate and configure EU868 for the end node. Once the end node is set up and the software is loaded onto the hardware, the TTN network configuration should include the following elements:

1. Frequency plan → 863–870 (SF11 for RX2).
2. Lorawan version → LoRaWAN Specification 1.0.2.
3. Regional Parameters version → RP001 Regional Parameters 1.0.2 revision B.
4. AppEUI → the configured LORAWAN_JOIN_EUI from the end node.
5. DevEUI → the configured LORAWAN_DEVICE_EUI from the end node.
6. AppKey → the configured LORAWAN_APP_KEY from the end node.

II. Gateway to Server: After receiving data from the devices, the LoRa gateways typically use another communication protocol, like Ethernet, Wi-Fi, or cellular, to forward this data to a central server. The server can be part of a cloud based LoRaWAN network or a private network, depending on the application.

The Gateway used is a NUCLEO-F746ZG board with a LRWAN_GS_HF1 shield [17], that can be observed in Figure 2 on the right side, being able to receive data from multiple nodes and to send it via ethernet to a server.

For the gateway configuration, the base software derived from ST Microcontroller was applied and loaded onto the Gateway. The subsequent action involved configuring the MAC address of the gateway to ensure it possesses a distinctive EUI address, as indicated on the hardware device. This address can be modified using the command: AT + MAC = 001122334455. Once the Gateway is set up with the updated software, the configuration for the TTN network should follow, requiring the configuration of the following elements:

1. Gateway EUI → Unique identifier that is configured for the Gateway.
2. Gateway Name → The name of the gateway that will be visible in TTN.
3. Gateway description → Description of the purpose of the Gateway.
4. Frequency plan → Europe 863–870 MHz (SF12 for RX2).

III. Server: The server, which can be a cloud-based platform or an on-premises server, collects data from multiple gateways and devices. It processes and stores these data, making it accessible for various applications and services. The server manages device registration, security, and data handling.

**Figure 3:**
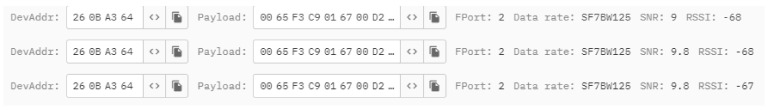
Data received by the server.

The Things Network serves as the server, allowing the visualization of received data from all configured end nodes through any device connected to the network. The data arrive in a payload, which the server decrypts, enabling the processing of the required information. Figure 3 illustrates this process, depicting the reception of information.

IV. Downlink Data: The server can also send commands or data back to specific LoRa devices through the gateways. This is known as “downlink” communication. The server communicates with the appropriate gateway, and the gateway then transmits the data or commands using LoRa to the target device.

The communication between the ST LoRa devices and gateways is primarily point to point, with the devices transmitting data when they need to. The gateways, on the other hand, are always listening for incoming LoRa transmissions. The server serves as the central hub for data collection, processing, and distribution [18,19].

It is important to note that this communication method is designed for low-power, long-range communication, making it suitable for applications like IoT (Internet of Things), smart cities, agriculture, and industrial automation, where devices need to transmit data over extended distances while conserving battery power.

In order to be able to set up the next steps and the grid of sensors to communicate with the gateway and transmit data received from the air quality sensors, a range test in a urban environment is needed to perform a test of the LoRaWAN protocol and the device limitations [20,21,22,23,24,25].

**Figure 4:**
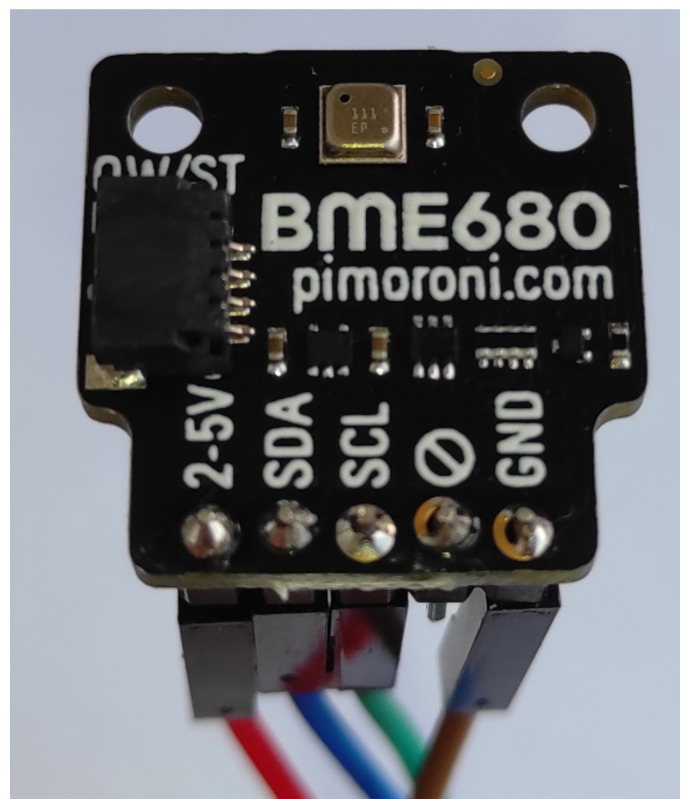
Pimorini BME680 Air Quality sensor.

The Sensor BME680 is used for air quality:

For the air quality measurement, the setup with the end node and the air quality sensor BME680 mounted on the breakout from Pimoroni [26] was established and can be observed in Figure 4.

The BME680 [27,28,29,30] is a gas sensor designed to measure various environmental parameters, including relative humidity, barometric pressure, ambient temperature, and gases (specifically VOCs, volatile organic compounds). It represents a pioneering advancement as the first gas sensor [31,32,33,34] to seamlessly integrate gas, pressure, humidity, and temperature sensors, offering exceptional linearity and accuracy. This sensor has been purposefully crafted for use in mobile applications and handheld devices, where compact size and minimal power consumption are crucial criteria.

In terms of performance, the BME680 ensures optimized power usage, long-term stability, and robust electromagnetic compatibility (EMC), depending on the selected operating mode. One of its notable capabilities is the ability to assess air quality for personal well-being, with the capacity to detect a wide array of gases, notably volatile organic compounds (VOCs).

## 3. Results

### 3.1. Setup and Concept

**Figure 5:**
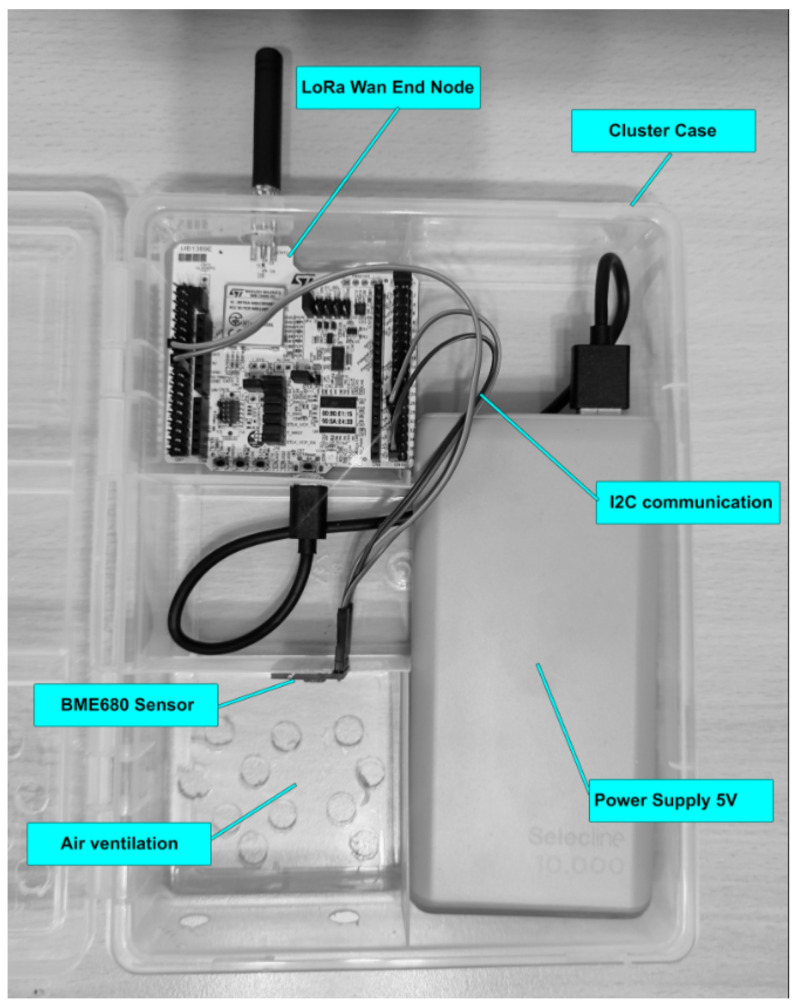
End node cluster.

**Figure 6:**
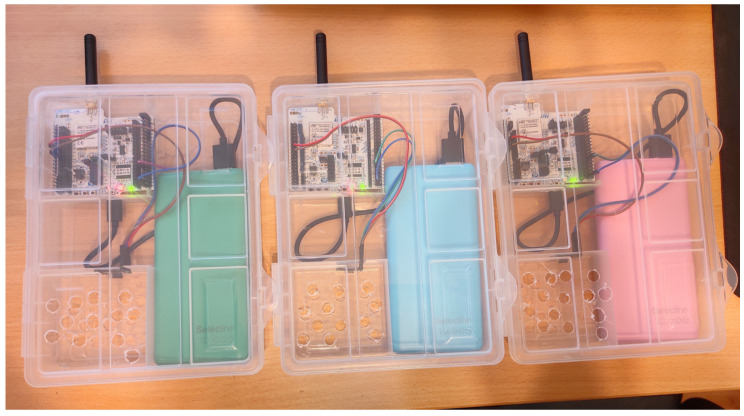
Multiple end node clusters.

To ensure the accuracy of the measurements, a cluster arrangement for the end node is essential, consolidating all components into a single case suitable for outdoor mounting to capture air quality data. The designed cluster, illustrated in Figure 5, serves this purpose. To collect precise data, multiple setups were required, as depicted in Figure 6.

To be mentioned that the current setup (cluster) is the first prototype, and this is designed just for the purpose of demonstrating the concept of having a mesh of air quality sensors around a crowded city.

The air quality sensor BME680 is connected to the end node via I2C communication, that means there are four wires needed to make the sensor functional: two wires for data transmission (SDA, serial data and SCL, serial clock) and two wires for power (GND and 2–5 V). An important configuration needs to be made in order to obtain the communication, and that is to set the I2C address for the current sensor to 0x77. After all the configurations are done the initialization part should be carefully configured and the steps can be analyzed in [20,21,22].

Once the end node cluster is set up, and all the necessary configuration and I2C communication between the AQI (Air Quality Index) sensor and the node are complete, the next step involves creating an effective pollution map. This entails defining the strategic placement of gateways and nodes to ensure comprehensive area coverage for measurements and to establish a clear line of sight between them to facilitate communication. As elucidated in reference [22], in an urban environment, particularly in the urban area of Cluj-Napoca city, direct line-of-sight communications were achieved spanning over 6000 m. This achievement is contingent on pollution levels and the establishment of a direct line of sight between the end node and the gateway.

In the initial setup, the end node transmitted the Payload containing all the information obtained and processed from the sensor to the gateway every 10 s. This rapid transmission rate led to a significant depletion of the battery. In our research paper, the measurements are taken every 30 s, but upon analysis, it appears that readings could be effectively acquired at intervals of 1 to 2 min, which would prove satisfactory. This more in-depth analysis of the optimal timing will be conducted in the subsequent development phase, following an examination of air fluctuations in various zones over a 24-h period.

The power usage of the end node, as provided by the manufacturer of the ST device, is as follows:

Based on the above measurements from Table 1, Table 2 and Table 3, if we take in discussing a battery of 2000–2500 mAh, for the first table (Table 1) of the power consumption for the setup with a wakeup time of 200 ms, sleep time 10 s, and uC working at 20 MHz, the battery will work for between 1.8 and 2.2 years. For the second table (Table 2) of the power consumption for the setup with a wakeup time of 200 ms, sleep time 30 s, and uC working at 80 MHz, the battery will work for between 3.2 and 3.9 years. For the third and last table (Table 3) of the power consumption for the setup with a wakeup time of 50 ms, sleep time 30 s, and uC working at 80 MHz, the battery will work for between 5.3 and 6.7 years. With the existing 10,000 mAh battery, it could run for about 20 years, provided that the battery’s power depletion over time does not occur [35].

For the measurements two scenarios are proposed:

1. The gateways and end nodes are elevated above the buildings. In this scenario, air quality monitoring can achieve with a reduced number of devices, primarily focusing on gateways. This approach ensures extensive coverage of a large area.
2. The gateways and end nodes are situated at an approximate elevation of 3–4 m above sidewalk level. This arrangement necessitates an increased number of gateways due to the higher positioning, which introduces more obstacles and results in significant signal attenuation. However, a notable advantage is that it provides pollution data that closely reflect the conditions experienced by people walking in that area. Placing the sensors on rooftop locations may expose them to air currents that circulate a greater volume of air, potentially yielding recorded values that differ from those at ground level.

Point 2, although more challenging to implement, serves as an illustrative example in a Cluj neighborhood, where the aim is to strategically position sensors and gateways for optimal effectiveness.

**Figure 7:**
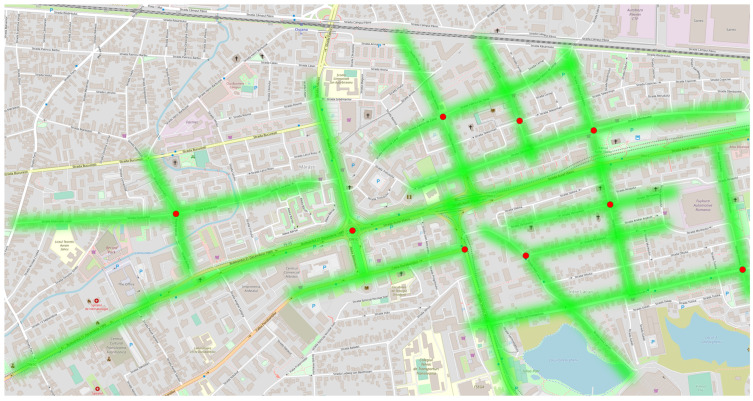
Gateways’ coverage of a neighborhood from Cluj-Napoca.

**Figure 8:**
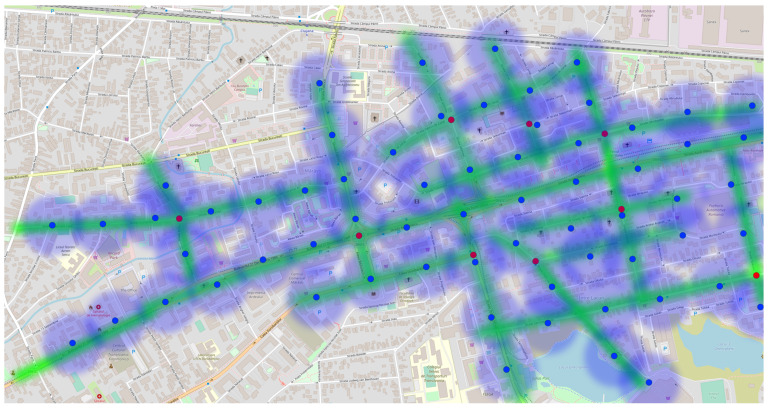
End node coverage of a neighborhood from Cluj-Napoca.

Figure 7 presents a solution tailored for densely populated urban areas. It suggests deploying nine gateways to achieve almost complete coverage of the designated zone. To ensure the precise monitoring of pollution levels throughout this extensive region, the recommended placing of the end node clusters is at intervals of 200 m. In this arrangement, for our illustrative scenario, a total of 59 sensors would be necessary to ensure comprehensive coverage of the entire area, as depicted in Figure 8.

**Figure 9:**
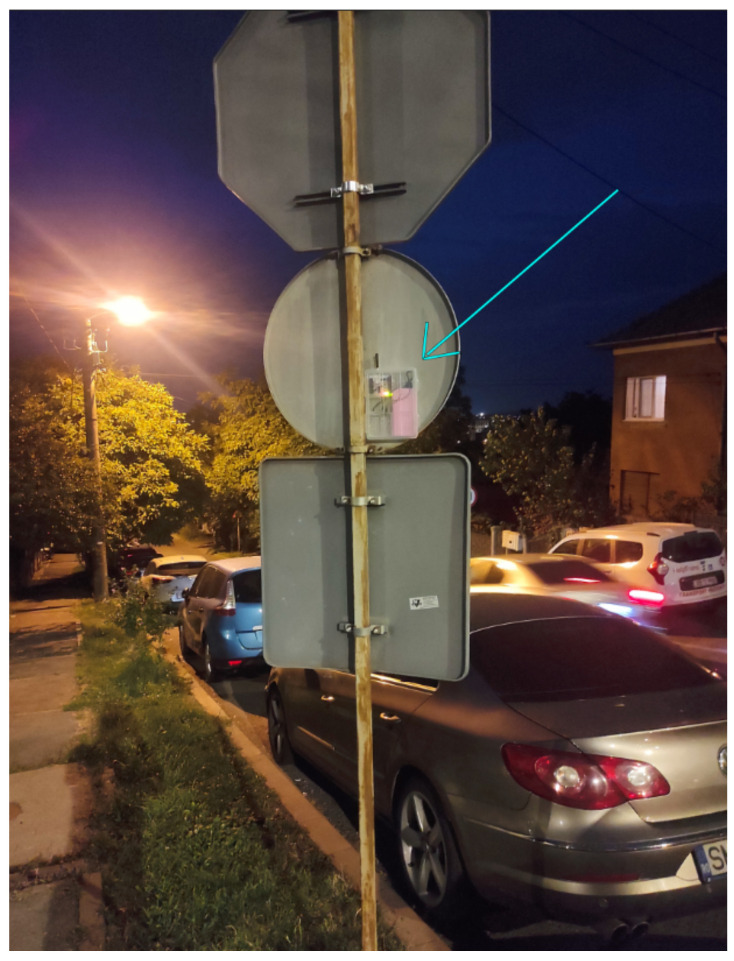
End node cluster mounted on signal pole (Highlighted by arrow).

Upon mounting all the end nodes (the mounted node is visible in Figure 9) and gateways, a real-time database can be established with the data received from them following the air quality measurements. With the existing setup, the conducted measurements lead to the obtained values presented in Table 1. These values were collected from three distinct areas to highlight significant differences. The BME sensors measured the following elements: barometric pressure, air quality resistance, humidity, and temperature.

The notable information is that, from the specification of the BME680 [29] sensor, the following information can be obtain visible in Table 4:

The table below displays the representation of gas (air quality) values, making it easier to assess the air quality in our surrounding area. The output of the BME680 sensor is a resistance value which can be converted into AQI values with the BME680 API provided as a library from the sensor manufacturer [35].

### 3.2. Measurements in Urban Area

In the following section, the air quality measurement was performed using designated end nodes, and the LoRa network was harnessed to transmit all recorded data to a server for centralized data collection. Equipped with AQI (Air Quality Index) sensors [36], these end nodes were strategically placed in three distinct zones within the city to facilitate the analysis and identification of varying pollution levels arising from factors such as heavy traffic, as well as different environmental conditions like fires, smoke, and dust resulting from construction activities, among others.

**Figure 10:**
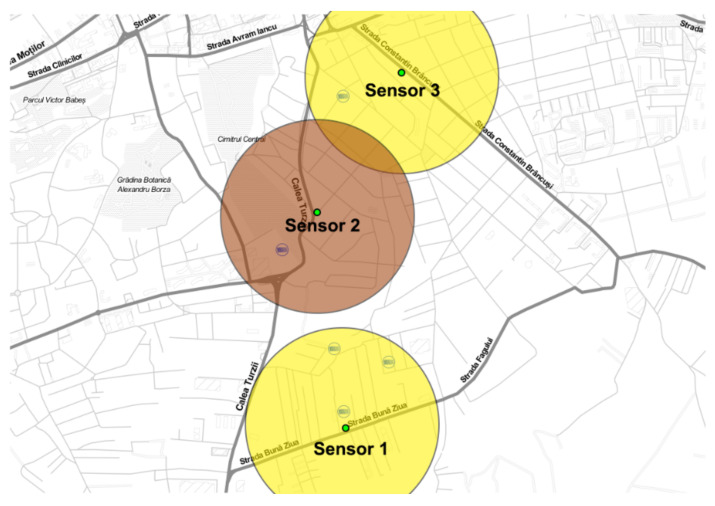
End nodes’ positions on the TTN mapper map (green dot) to be in the range of a gateway for data transmission.

The positioning of these nodes within the city was based on the availability of existing gateways in the urban area. To ascertain gateway locations and their operational status, reference was made to the TTN mapper web page, which offers accurate locations and status updates for gateways in the current region. Following this, the gateway positions were mapped out, and the locations of the end nodes were added to the same map, as depicted in Figure 10.

For each sensor, the proposal is that it can offer an accurate air quality measurement for an area within a 500-m radius. Each node is mapped on the map with a green dot.

Three distinct tables were used for the measurements to facilitate a thorough comparison of the acquired data, with particular emphasis on the gas measurements. This emphasis on gas measurements stems from the fact that pressure, humidity, and temperature readings exhibited only minor fluctuations, owing to the relatively limited range of the devices.

**Figure 11:**
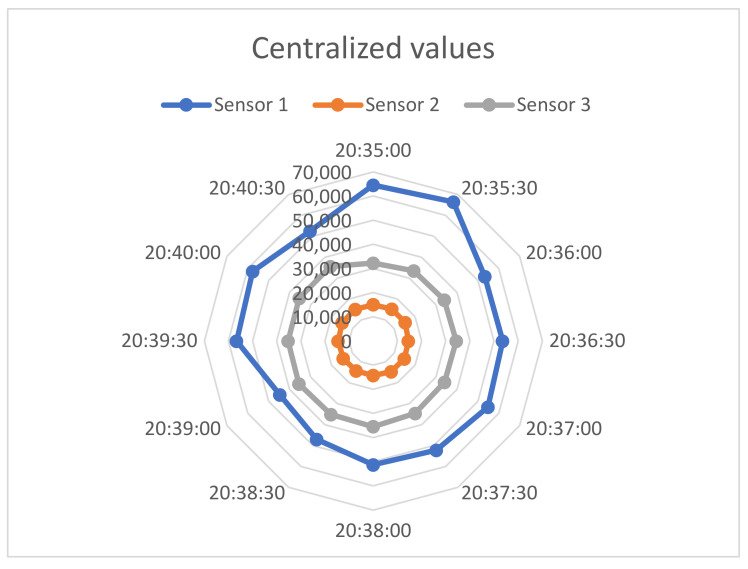
Diagram of the centralized values for gas measurement from sensors 1, 2, and 3.

Our method centered on employing the initial table as a reference point for assessing gas measurement values presented in Table 5. Following this, by integrating data from Table 6, Table 7 and Table 8, Figure 11 was constructed, which offered a straightforward interpretation of gas measurements, rendering them comprehensible in the context of air quality conditions.

By utilizing the determined status of each node, specific colors were assigned to depict the sensor area diameters. For instance, yellow was allocated to denote an area with average pollution for nodes one and three, while brown was used to signify a zone with slightly elevated pollution levels for the second node.

### 3.3. Usage of the Air Pollution Map

Building upon the preceding subsection, in which a successfully measured air pollution in three distinct city areas was performed and having the result visualized on a map (Figure 10) using various colors (with Table 5 as a reference and Figure 11 as the measured values), a strategy can now be formulated a strategy to navigate from point A to point B while optimizing for the cleanest air quality within a specific time frame. To illustrate this concept, the same pollution map from Figure 10 was employed, with two points marked as red dots to symbolize the starting and ending locations. These points allowed us to generate multiple possible routes, a common feature in existing traffic applications where one can typically choose the fastest or shortest route. However, what is currently lacking in infrastructure is the ability to select the cleanest route, as real-time air quality data is not readily available.

**Figure 12:**
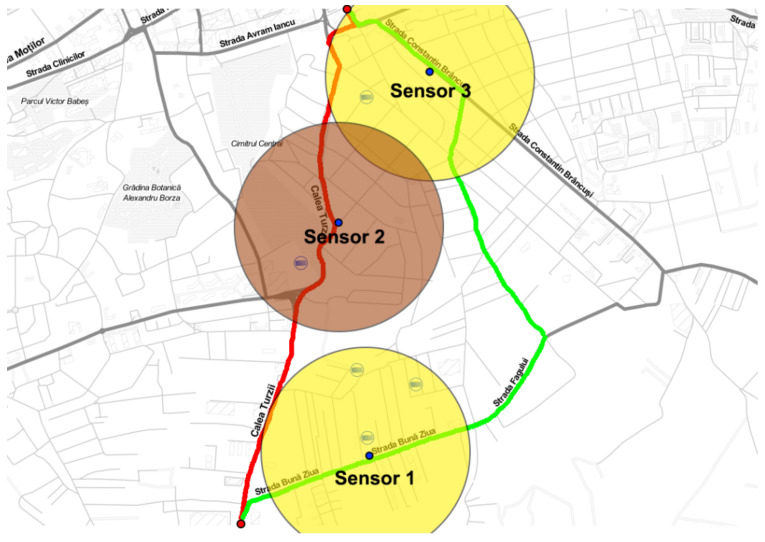
Air pollution map and possible routes with different pollution levels (Red dot—start and destination point, and blue dot—the location of the end nodes).

The ongoing project has the objective of bridging this void through the implementation of a real-time Air Quality Index (AQI) map, allowing the integration of air quality data into traffic coordination. In this particular instance, two potential routes were identified, as depicted in Figure 12, with each presenting different pollution levels. Notably, sensor 2 detected higher pollution levels than sensors 1 and 3. Consequently, the green path emerges as the preferred route due to its lower pollution levels. A closer examination reveals that the red path traverses all three pollution zones, making it the most polluted route.

Drawing from this prototype implementation, potential enhancements could positively impact the quality of life for urban residents where pollution levels often surpass the acceptable or safe thresholds. Many individuals might traverse highly polluted areas without awareness that there could be an alternative, safer route to their destination.

In order to determine a viable route, it is essential to incorporate a route selection algorithm [36], which, in this case, will use the pollution data as input to identify the route with the lowest pollution levels. The algorithms and techniques for computing optimal trajectories [37] have already been established and will not be the primary focus of this paper. The primary objective of the current paper is the implementation of a pollution map to gain insights into pollution levels and their significant variations within small geographical areas.

As part of future research and development, the implemented pollution map will incorporate an algorithm designed to compute the optimal path between two points based on pollution levels.

## 4. Discussion

In the previous section, it was effectively demonstrated that, even with just three strategically placed sensors within a city area, noticeable variations in pollution levels are evident. This finding highlights the limitations of relying on only two or a maximum of three air quality monitoring stations to construct an accurate pollution map. Pollution levels can fluctuate significantly every 200 m, influenced by factors like geographical terrain, prevailing air currents, and pollution sources.

To achieve a precise and comprehensive pollution map, the implementation of a sensor mesh network, as explained in Section 3.1 (as shown visually in Figure 8), becomes essential. With the deployment of a dense network of air quality sensors across a city area, precise identification of pollution incidents is possible. Moreover, this improved sensor infrastructure equips authorities to proactively intervene before unhealthy air quality can spread throughout an entire neighborhood.

In relation to the setup costs, the current cluster functions as a prototype designed to facilitate data measurements and showcase the necessary sensor infrastructure. To provide more detailed information, the total cost of this setup amounts to approximately 82 Euros. This cost includes the following components: a 10,000 mAh battery, a plastic casing, a BME680 sensor, an end node, and a portion of the gateway cost. It is important to note that the gateway cost should not be solely assigned to the total cost of each individual device. Instead, it should be distributed among the medium devices connected to it, with an average distribution of about 20 devices.

As for the existing cluster, it is crucial to highlight that its dimensions are not conducive to integration within a complex and densely populated urban environment. The current dimensions of the end node setup measure 150 × 200 × 30 mm.

Looking ahead to future endeavors, there is an intention to design dedicated hardware with the aim of achieving a smaller form factor, approximately 40 × 40 × 30 mm, and reducing the cost per device to around 20 Euros. If these objectives are successfully achieved, the implementation of the sensor mesh within a city will become significantly more manageable.

Entering deeper into the discussion of the importance of this proposed paper and the advantages of this proposed idea, one must consider the context of a large and densely populated city. Presently, such cities typically feature between 3 and 10 air quality stations. The goal is to predict the overall pollution levels across the entire city using the existing equipment. This current setup relies heavily on extensive predictions. In the best-case scenario, it offers limited relevance to the entire city’s expanse.

To provide a direct analogy, having only three air quality stations in a city and declaring “the pollution of that city is…” based solely on these three measurements is akin to asserting that a city has citywide wireless internet coverage because there are three wireless routers in the city center, and due to these three access points, anyone is able to connect to the internet from any zone of the city.

## 5. Conclusions

Drawing upon the findings of our study, it is essential to recognize that, in a bustling and densely populated city like Cluj-Napoca, pollution levels cannot be reliably predicted based solely on measurements taken at a mere three locations encompassing the entire city, where the current air quality monitoring stations are installed. Hence, there is an imperative need to design and deploy a mesh network of Air Quality Index (AQI) sensors, ensuring that these sensors are situated within a proximity of no more than 200 m from one another to construct an accurate pollution map.

These Air Quality Index sensors should ideally employ LoRaWAN technology to facilitate the transmission of compact data packets over extended distances. While this approach may not reduce the number of end nodes required for generating the pollution map, it does streamline the infrastructure requirements, meaning a reduced number of gateways with an internet connection will be necessary for relaying all received data to a central server for processing.

An important consideration is that the strategic placement of gateways and end nodes must be meticulously planned to maximize the number of end nodes capable of communicating with a single gateway. This approach can enable communication distances of up to 7000 m, contingent upon having an unobstructed line of sight between the devices.

Once the entire sensor mesh is deployed in a designated area, a real-time pollution map can be generated, providing individuals with immediate access when deciding where to go for a walk, take an electric scooter ride, or embark on a bicycle trip. This gives users the ability to choose areas with optimal air quality for their specific plans.

Furthermore, these data can be smoothly incorporated into navigation applications, allowing users to plan their routes between two selected addresses within the city based on air quality, even if it involves selecting a longer route or a slightly extended travel time.

The only prediction to consider is that if a pollution source exists within the city with elevated pollution levels, it becomes evident that pollution levels will diminish as you move from the central point to the outskirts until you reach an area with notably lower pollution levels or until another pollution reading is encountered. When another sensor is encountered, an average reading can be established if the distance between them is no greater than 500–600 m.

Based on our comprehensive analysis, it is worth noting that the implementation of the mentioned pollution map by city authorities can potentially lead to a significant reduction in pollutants during the initial months after deployment. This system empowers authorities to identify sources of city pollution that were previously invisible to the naked eye or undetectable with portable Air Quality Index (AQI) sensors.

The limitations of portable sensors lie in their extended calibration periods and the possibility of being already some distance away from the pollution source while in motion. This can result in misdirected efforts and delays in taking corrective action. Additionally, covering the entire city simultaneously with portable devices within the same timeframe is often impractical.

## Figures and Tables

**Table 1:** Table of the power consumption for the setup with a wakeup time of 200 ms, sleep time 10 s, and uC working at 20 MHz.

Status	[mA] × [s]	T_Cycle
Stop2 (+RTC) mode: 1.07 µA (VDD = 3 V)	0.010	10 s–0.2 s
Active-mode MCU: <72 µA/MHz (CoreMark^®^)	0.306	200 ms
Active-mode RX: 4.82 mA	0.096	20 ms
Active-mode TX: 87 mA at 20 dBm (LoRa^®^ 125 kHz)	0.870	10 ms
I sensor ULP mode: 90 µA (Avrage)		
I_average	0.128	mA
Battery power per year	**1123.81**	**mAh**

**Table 2:** Table of the power consumption for the setup with a wakeup time of 200 ms, sleep time 30 s, and uC working at 80 MHz.

Status	[mA] × [s]	T_Cycle
Stop2 (+RTC) mode: 1.07 µA (VDD = 3 V)	0.032	30 s–0.2 s
Active-mode MCU: <72 µA/MHz (CoreMark^®^)	1.170	200 ms
Active-mode RX: 4.82 mA	0.096	20 ms
Active-mode TX: 87 mA at 20 dBm (LoRa^®^ 125 kHz)	0.870	10 ms
I sensor ULP mode: 90 µA (Avrage)		
I_average	0.072	mA
Battery power per year	**633.14**	**mAh**

**Table 3:** Table of the power consumption for the setup with a wakeup time of 50 ms, sleep time 30 s, and uC working at 80 MHz.

Status	[mA] × [s]	T_Cycle
Stop2 (+RTC) mode: 1.07 µA (VDD = 3 V)	0.032	30 s–0.05 s
Active-mode MCU: <72 µA/MHz (CoreMark^®^)	0.293	50 ms
Active-mode RX: 4.82 mA	0.096	20 ms
Active-mode TX: 87 mA at 20 dBm (LoRa^®^ 125 kHz)	0.870	10 ms
I sensor ULP mode: 90 µA (Avrage)		
I_average	0.043	mA
Battery power per year	**376.96**	**mAh**

**Table 4:** Element table displaying their attainable value ranges.

Element	Min. Value	Max Value	Unit
Pressure	300	1100	hPa
Temperature	−40	85	°C
Humidity	10	90	% R.H
Gas	244	8,000,000	Ohm

**Table 5:** Table of the values (resistance in Ohms) output by the device.

Gas Level	Constant Range (Ohms)	Status
0	8,000,000–4,000,001	Very good
1	4,000,000–2,000,001	Very good
2	2,000,000–1,000,001	Very good
3	1,000,000–499,501	Good
4	499,500–248,263	Good
5	248,262–125,001	Good
6	125,000–63,005	Average
7	63,004–31,282	Average
8	31,281–15,626	Little Bad
9	15,625–7813	Little Bad
10	7812–3907	Bad
11	3906–1954	Bad
12	1953–977	Worse
13	976–489	Worse
14	488–245	Very Bad
15	244	Very bad

**Table 6:** Table of the values measured by sensor nr.1.

Sensor 1	Pressure	Temperature	Humidity	Gas Measurement
20:35:00	964	21	62	64,531
20:35:30	965	22	61	66,351
20:36:00	965	22	61	53,254
20:36:30	965	22	60	53,553
20:37:00	964	22	60	54,748
20:37:30	964	22	60	52,231
20:38:00	964	22	59	51,211
20:38:30	964	22	57	47,024
20:39:00	965	21	57	44,642
20:39:30	964	22	57	56,642
20:40:00	964	22	57	57,661
20:40:30	964	22	56	52,467

**Table 7:** Table of the values measured by sensor nr.2.

Sensor 2	Pressure	Temperature	Humidity	Gas Measurement
20:35:00	965	21	59	15,023
20:35:30	965	21	59	15,198
20:36:00	964	21	57	15,207
20:36:30	965	22	57	14,503
20:37:00	965	22	58	14,787
20:37:30	965	22	59	14,691
20:38:00	965	21	59	14,263
20:38:30	965	21	59	14,314
20:39:00	966	21	59	14,427
20:39:30	966	22	57	14,587
20:40:00	966	21	57	14,921
20:40:30	966	21	57	15,084

**Table 8:** Table of the values measured by sensor nr.3.

Sensor 3	Pressure	Temperature	Humidity	Gas Measurement
20:35:00	967	21	58	32,241
20:35:30	967	22	58	33,546
20:36:00	968	22	57	33,975
20:36:30	968	22	57	34,321
20:37:00	968	22	57	34,092
20:37:30	968	23	57	34,671
20:38:00	968	22	58	35,449
20:38:30	969	22	59	35,217
20:39:00	969	22	59	35,521
20:39:30	969	22	59	35,318
20:40:00	968	22	58	35,443
20:40:30	968	22	58	35,533

## Data Availability

Not applicable.

## References

[1] Sarroeira R., Henriques J., Sousa A.M., Ferreira da Silva C., Nunes N., Moro S., Botelho M.d.C. (2023). Monitoring Sensors for Urban Air Quality: The Case of the Municipality of Lisbon. Sensors.

[2] Messaoud T.A., Smiti A. The COVID-19 Pandemic: What About Air Pollution?. Proceedings of the 2020 4th International Conference on Advanced Systems and Emergent Technologies (IC_ASET).

[3] Alkindi F., Al R., Shanableh A., Hamdan H.A. Integrate Ground Data and Remote Sensing to Monitor SO_2_ and NO_2_ Pollution Over Northern UAE/Considering the Lockdown Period of the COVID-19 Pandemic. Proceedings of the IGARSS 2022—2022 IEEE International Geoscience and Remote Sensing Symposium.

[4] Nandanwar H., Chauhan A. Comparative Study of Impact of COVID-19 Lockdown on AQI Parameters Across Urban India. Proceedings of the 2021 International Conference on Smart Generation Computing, Communication and Networking (SMART GENCON).

[5] Idir Y.M., Orfila O., Judalet V., Sagot B., Chatellier P. (2021). Mapping Urban Air Quality from Mobile Sensors Using Spatio-Temporal Geostatistics. Sensors.

[6] Garg K.D., Gupta M., Sharma B., Dhaou I.B. A Comparison of Regression Techniques for Prediction of Air Quality in Smart Cities. Proceedings of the 2023 1st International Conference on Advanced Innovations in Smart Cities (ICAISC).

[7] Egodagama W.G.C.N., Zahid A.A.M., Pathirana G.P.T.S., Chathurika B., Supunya R. Air Pollution Mapping with Sensor-based Methodology. Proceedings of the 2021 3rd International Conference on Advancements in Computing (ICAC).

[8] Munir S., Mayfield M., Coca D., Jubb S.A. (2019). Structuring an integrated air quality monitoring network in large urban areas—Discussing the purpose, criteria, and deployment strategy. Atmos. Environ. X.

[9] O’Kennedy M., Niesler T., Wolhuter R., Mitton N. Practical evaluation of carrier sensing for a LoRa wildlife monitoring network. Proceedings of the 2020 IFIP Networking Conference (Networking).

[10] Nilesh N., Narang J., Parmar A., Chaudhari S. IoT and ML-based AQI Estimation using Real-time Traffic Data. Proceedings of the 2022 IEEE 8th World Forum on Internet of Things (WF-IoT).

[11] Zhang H., Song Y., Yang M., Jia Q. (2023). Modeling and Optimization of LoRa Networks under Multiple Constraints. Sensors.

[12] LoRa Alliance—About Lora. https://lora-alliance.org/about-lora-alliance/.

[13] Dragulinescu A.-M., Halunga S., Zamfirescu C. (2021). Unmanned Vehicles’ Placement Optimisation for Internet of Things and Internet of Unmanned Vehicles. Sensors.

[14] Chaudhari P., Tiwari A.K., Pattewar S., Shelke S.N. Smart Infrastructure Monitoring using LoRaWAN Technology. Proceedings of the 2021 International Conference on System Computation, Automation and Networking (ICSCAN).

[15] LoRa Alliance Operational Documents. LoRa Alliance Confidentiality & Communications Policy. https://hz137b.p3cdn1.secureserver.net/wp-content/uploads/2021/05/LoRa-Alliance-Confidentiality-and-Communications-Policy-Approved-07.02.20.pdf.

[16] SamTech LoRa-Why Lora. SamTech. [Interactiv]. https://www.semtech.com/lora/why-lora.

[17] ST Microcontroller—Nucleo-WL55JC1. https://www.st.com/en/evaluation-tools/nucleo-wl55jc.html.

[18] Kostadinov A., Kolev K. LoRa Smart City Applications. Proceedings of the 2022 29th International Conference on Systems, Signals and Image Processing (IWSSIP).

[19] P-Nucleo-LRWAN2. https://www.st.com/resource/en/user_manual/um2587-getting-started-with-the-pnucleolrwan2-and-pnucleolrwan3-starter-packs-stmicroelectronics.pdf.

[20] IoDobra M., Dobra V.A., Gavra V.D., Folea S. LoRa propagation test in an urban environment and office buildings. Proceedings of the International IEEE AQTR Conference.

[21] Jörke P., Böcker S., Liedmann F., Wietfeld C. Urban channel models for smart city IoT-networks based on empirical measurements of LoRa-links at 433 and 868 MHz. Proceedings of the 2017 IEEE 28th Annual International Symposium on Personal, Indoor, and Mobile Radio Communications (PIMRC).

[22] IoDobra M., Dobra A.A., Dobra V.A., Gavra V.D., Folea S. Air Quality Analysis in the Surrounding Environments Using a Lora Network. Proceedings of the 2023 27th International Conference on Production Research.

[23] Horstmann T., Rademacher M., Roobi M., Weckmann S. Evaluation of LoRa in a Real-World Smart City: Selected Insights and Findings. Mobile Communication—Technologies and Applications. Proceedings of the 27th ITG-Symposium.

[24] Sundaram J.P.S., Du W., Zhao Z. (2020). A Survey on LoRa Networking: Research Problems, Current Solutions, and Open Issues. IEEE Commun. Surv. Tutor..

[25] Sağır S., Kaya İ., Şişman C., Baltacı Y., Ünal S. Evaluation of Low-Power Long Distance Radio Communication in Urban Areas: LoRa and Impact of Spreading Factor. Proceedings of the 2019 7th International Conference on Digital Information Processing and Communications (ICDIPC).

[26] Pimoroni BME680 Breakout. https://shop.pimoroni.com/products/bme680-breakout?variant=12491552129107.

[27] Bosch BME680. https://www.bosch-sensortec.com/products/environmental-sensors/gas-sensors/bme680/.

[28] Bosch BME688, Specification. https://www.bosch-sensortec.com/media/boschsensortec/downloads/product_flyer/bst-bme688-fl001.pdf.

[29] Bosch BME688. https://www.bosch-sensortec.com/products/environmental-sensors/gas-sensors/bme688/.

[30] Sântejudean T.V., Mois G.D., Sanislav T., Folea S.C. (2022). Edge Computing in Wireless Sensing Applications. Proceedings of the 2022 11th Mediterranean Conference on Embedded Computing (MECO).

[31] Folea S.C., Mois G.D. (2019). Lessons learned from the development of wireless environmental sensors. IEEE Trans. Instrum. Meas..

[32] Sherazi H.H.R., Grieco L.A., Imran M.A., Boggia G. (2021). Energy-Efficient LoRaWAN for Industry 4.0 Applications. IEEE Trans. Ind. Inform..

[33] Kadir A.D.I.A., Alias M.R.N.M., Dzaki D.R.M., Din N.M., Deros S.N.M., Haron M.H. Cloud-Based IoT Air Quality Monitoring System. Proceedings of the 2021 26th IEEE Asia-Pacific Conference on Communications (APCC).

[34] Park H., Lakshminarayana S., Nguyen L.H.T., Pan C., Jung S. Portable Indoor Air Quality Measurement System. Proceedings of the 2022 E-Health and Bioengineering Conference (EHB).

[35] Bosch BME680—Data Sheet. https://www.bosch-sensortec.com/media/boschsensortec/downloads/datasheets/bst-bme680-ds001.pdf.

[36] Armond A.M., Prasetyo Y.D., Ediningrum W. Application of Ant Colony Optimization (ACO) Algorithm to Optimize Trans Banyumas Bus Routes. Proceedings of the 2022 IEEE International Conference on Cybernetics and Computational Intelligence (CyberneticsCom).

[37] Sun J., Yu Y., Xin L. Research on Path Planning of AGV Based on Improved Ant Colony Optimization Algorithm. Proceedings of the 2021 33rd Chinese Control and Decision Conference (CCDC).

